# Immune dysregulation and postoperative wound complications in systemic autoimmune rheumatic diseases: toward disease-specific risk stratification

**DOI:** 10.3389/fimmu.2026.1794700

**Published:** 2026-07-31

**Authors:** Shiuan-Tzuen Su, James C.-C. Wei

**Affiliations:** 1 Department of Surgery, Chung Shan Medical University Hospital, Taichung, Taiwan; 2 School of Medicine, Chung Shan Medical University, Taichung, Taiwan; 3 Institute of Medicine, Chung Shan Medical University, Taichung, Taiwan; 4 Division of Allergy, Immunology and Rheumatology, Department of Internal Medicine, Chung Shan Medical University Hospital, Taichung, Taiwan; 5 Graduate Institute of Integrated Medicine, China Medical University, Taichung, Taiwan

**Keywords:** autoimmune risks, immunomodulatory, impaired wound healing, inflammation and inflammatory markers, surgical site infection, wound complications, wound dehiscence, wound healing

## 1 Immune dysregulation and impaired wound healing in autoimmune diseases

Despite increasing recognition that systemic autoimmune rheumatic diseases are associated with poor postoperative wound outcomes, current perioperative risk assessment remains largely based on conventional surgical variables and largely overlooks the biological consequences of immune dysregulation. We propose that immune dysfunction should serve as the organizing principle for disease-specific risk stratification because it integrates inflammatory activity, vascular injury, tissue repair capacity, and immunomodulatory therapy into a unified mechanistic framework. This Opinion argues that an immunology-centered model may better explain postoperative wound vulnerability than existing surgery-centered approaches. Accordingly, we advocate that future autoimmune-specific risk models should prioritize immune activity, vascular injury, and immunomodulatory exposure as primary determinants of postoperative wound vulnerability, with conventional surgical variables serving only complementary roles.

Autoimmune diseases (ADs) have represented a heterogeneous group of systemic immune-mediated disorders characterized by persistent activation of innate and adaptive immune pathways. Loss of immune tolerance led to autoantibody production, immune-complex deposition, complement activation, and sustained cytokine release, including tumor necrosis factor-α (TNF-α), interleukin-6 (IL-6), interferons, and other pro-inflammatory mediators. From a wound-healing perspective, this chronic inflammatory state fundamentally altered the normal biologic cascade required for tissue repair (1, 2).

Physiologic wound healing occurs through four regulated stages hemostasis, inflammation, proliferation, and remodeling (3). During the inflammatory phase of wound healing (24–48 h after injury), innate and adaptive immune cells coordinate host defense and tissue repair. Keratinocytes detect pathogen- and danger-associated signals through pattern-recognition receptors such as Toll-like receptors, activating Nuclear factor kappa-light-chain-enhancer of activated B cells (NF-κB) and mitogen-activated protein kinase (MAPK) pathways to produce cytokines and chemokines that recruit leukocytes (4). Neutrophils eliminate pathogens and necrotic debris through phagocytosis, reactive oxygen species (ROS), and proteolytic enzymes, whereas macrophages orchestrate the transition from inflammation toward tissue repair. Successful healing requires macrophage transition from M1 to M2 phenotypes, which promotes angiogenesis, extracellular matrix (ECM) remodeling, fibroblast activation, and re-epithelialization (5, 6).

Adaptive immune cells and dendritic cells further modulate epithelial regeneration and inflammatory balance (4). Immunomodulation plays a central role throughout this process. Regulatory T cells (Tregs) are increasingly recognized as key mediators of tissue homeostasis and regenerative healing. Tregs suppress excessive inflammation through anti-inflammatory cytokines such as IL-10 and transforming growth factor-β (TGF-β), while also supporting vascularization, fibroblast regulation, and epithelial repair (7). In parallel, endothelial protein C receptor (EPCR) signaling contributes to cytoprotection, vascular homeostasis, and anti-inflammatory regulation by modulating immune-cell activity and stabilizing endothelial barriers. EPCR additionally promotes endothelial and epithelial proliferation, neovascularization, and tissue remodeling through activation of protein C and protease-activated receptor signaling pathways (8). Chemokine pathways such as stromal-derived factor-1 (SDF-1/CXCL12) further regulate progenitor-cell recruitment, angiogenesis, and regenerative signaling, linking immunology with regenerative medicine and biomaterial-based therapies (9). Multiple intracellular signaling pathways also coordinate immune and stromal-cell responses during wound healing. G-protein-coupled receptors (GPCRs) regulate immune-cell recruitment, keratinocyte proliferation, and fibroblast activation through pathways including Hedgehog-GLI, Hippo-YAP1, and Wnt/β-catenin signaling (10). These pathways may integrate inflammatory signals with mechanotransduction and ECM remodeling, thereby influencing scar formation and tissue regeneration. However, persistent immune activation may disrupt this tightly regulated balance. Type 17 immunity and IL-17 signaling demonstrate a “double-edged” role in wound repair. Acute IL-17 activity promotes antimicrobial defense and early neutrophil recruitment, whereas sustained IL-17 overexpression prolongs inflammation, impairs keratinocyte migration and angiogenesis, and contributes to chronic non-healing wounds (11).

In ADs, wounds frequently failed to transition beyond the inflammatory phase, resulting in excessive proteolytic activity, impaired fibroblast migration, dysfunctional angiogenesis, and delayed collagen deposition. Evidence from tertiary wound care cohorts have demonstrated that chronic wounds in patients with rheumatoid arthritis (RA), systemic lupus erythematosus (SLE), vasculitis, and other connective tissue diseases (CTDs) exhibited markedly prolonged healing times compared with non-autoimmune populations (12).

Vascular involvement further compounded this risk. Vasculitic and immune-mediated endothelial injury led to microthrombosis, ischemia-reperfusion injury, and impaired oxygen delivery, all of which directly compromised wound viability. Studies focusing on chronic limb ischemia and ADs illustrated how immune-mediated inflammation produced a pro-atherothrombotic phenotype that adversely affected both acute and chronic wound outcomes (13).

In ADs, postoperative wound complications frequently arise from systemic immune dysregulation rather than local surgical factors alone. Chronic inflammation, endothelial dysfunction, vasculopathy, abnormal cytokine production, impaired Treg activity, persistent M1 macrophage polarization, and dysregulated Th17 responses collectively interfere with normal wound progression. Furthermore, excessive ROS production, matrix metalloproteinases (MMPs) activation, microbiome imbalance, and defective ECM remodeling may establish a self-perpetuating inflammatory cycle that delays wound closure and promotes fibrosis or chronic ulceration (6). Emerging evidence suggests that biomarkers such as IL-6, IL-10, TNF-α, MMPs, vascular endothelial growth factor (VEGF), TGF-β1, and platelet-derived growth factor (PDGF) may help predict wound-healing outcomes and identify high-risk patients (14). Mast cells may contribute to postoperative wound complications in systemic autoimmune rheumatic diseases by linking immune dysregulation, inflammation, vascular permeability, fibrosis, and tissue remodeling. After tissue injury, mast cells rapidly release histamine, tryptase, chymase, TNF-α, IL-6, IL-8, VEGF, PDGF, and TGF-β, thereby promoting leukocyte recruitment, angiogenesis, fibroblast activation, collagen deposition, and re-epithelialization. However, their role is context-dependent. Appropriate activation may support early host defense and repair, whereas excessive or persistent mast cell activation may amplify inflammation, fibrosis, scar formation, or delayed healing. Therefore, mast cells may serve as disease-specific immune regulators and biomarkers for postoperative wound risk stratification in autoimmune rheumatic diseases (15–17). Collectively, these findings support an immunology-centered framework for disease-specific risk stratification and perioperative optimization in patients with ADs.

## 2 Postoperative wound complications in patients with autoimmune diseases

Across surgical disciplines, ADs have associated with increased risk of postoperative complications. Large database studies, propensity-matched cohorts, and systematic reviews have consistently reported increased risks of surgical-site infection (SSI), wound dehiscence, delayed healing, thromboembolic events, and reoperation. For patients with inflammatory bowel disease (IBD), a simple risk score for SSI after bowel resection incorporated preoperative and intraoperative variables such as weight loss, smoking, emergent surgery, wound class, operative time, and ASA score >2. This score stratified patients into low, medium, and high risk for SSI, with increasing rates of complications across these categories (18). However, no validated model currently provides optimal predictive accuracy for postoperative wound complications in patients with ADs. **Table 1** summarizes existing clinical studies addressing postoperative wound outcomes in this population.

**Table 1:** Postoperative outcomes in patients with autoimmune diseases undergoing surgical procedures.

Author, year	Participants, n	Disease context	Medication exposure	Procedure	Postoperative outcomes	Study design/level of evidence
Chang et al., 2025 (19)	448	AD	—	CTR	Functional improvement observed; increased risks of SSI, prolonged edema, and revision surgery compared with non-rheumatic controls.	Retrospective/Level II
Madelar et al., 2023 (21)	386	AD	—	ASD	Higher rates of systemic medical complications and longer hospitalization; radiographic alignment remained similar.	Retrospective/Level II
Blizzard et al., 2017 (22)	1006	AS	—	THA	Increased hip dislocation, periprosthetic fracture, wound complications, revision THA.	Retrospective/Level II
Rubio et al., 2018 (23)	537	CTD	—	Abdominoplasty	Feasible, but associated with increased risks of VTE and blood transfusion	Retrospective/Level II
Li et al., 2023 (24)	12	AD	—	OGS	Delayed neurosensory recovery; higher rates of infection and TMJ complications.	Retrospective/Level II
Fung et al., 2025 (25)	1290	AD	DMARDs	Implant-based breast reconstruction	Higher rates of necrosis and surgical site pain within 90 days; increased infection, DVT, PE, ER visits, capsulectomy; higher capsular contracture among conventional DMARDs compared with bDMARDs users.	Retrospective/Level II
Tada et al., 2016 (26)	227	RA	bDMARDs	Elective orthopedic surgery	bDMARDs did not increase SSI risk; foot surgery delayed wound healing	Retrospective/Level II
O’Brien-Irr et al., 2019 (33)	297	CIID	Steroids or anti-inflammatory agents	Lower extremity amputations or revascularization	Limb salvage rates comparable to those of patients without IMD; steroid or anti-inflammatory use beneficial.	Retrospective/Level II
Herrera Van Oostdam et al., 2023 (34)	53	RD	—	Major surgery	Higher infection rates, uncontrolled hypertension, and acute preoperative kidney injury predicted postoperative complications.	Retrospective/Level II

AD, autoimmune disease; CTR, Carpal tunnel release; SSI, surgical site infection; ASD, adult spinal deformity; AS, Ankylosing spondylitis; THA, total hip arthroplasty; CTD, Connective Tissue Disease; VTE, venous thromboembolism; OGS, orthognathic surgery; TMJ, temporomandibular joint; DMARDs, disease-modifying antirheumatic drugs; DVT, Deep Vein Thrombosis, PE, pulmonary embolism, ER, emergency room; bDMARDs, Biological disease-modifying antirheumatic drugs; RA, rheumatoid arthritis; CIID, Chronic immune-mediated inflammatory disease; IMD, immune-mediated disease; RD, rheumatic diseases.

In hand surgery, patients with ADs undergoing carpal tunnel release experienced nearly fourfold higher overall complication rates compared with non-rheumatic controls, including increased infection, prolonged edema, and revision surgery, despite comparable functional improvement (19). Orthopedic studies further reinforced the elevated surgical risk in autoimmune populations. Meta-analyses of spinal surgery have demonstrated that RA significantly increased postoperative infection and overall complication rates, with pooled odds ratios exceeding 1.5 for both outcomes (20). Similarly, patients with ADs undergoing adult spinal deformity surgery experienced higher medical complications and worse patient-reported outcomes despite comparable radiographic alignment (21). In ankylosing spondylitis (AS), the combination of altered biomechanics and systemic inflammation resulted in increasing rates of periprosthetic fracture, dislocation, and revision following total hip arthroplasty (22). In aesthetic and reconstructive surgery, ADs were associated with higher rates of wound complications and venous thromboembolism following abdominoplasty, indicating that increased risk extended beyond traditionally high-risk procedures (23). Likewise, orthognathic surgery series report elevated rates of postoperative infection, neurosensory deficits, and temporomandibular joint complications in patients with ADs, highlighting the systemic surgical vulnerability of this population (24).

The consistency of risk underscores the importance of comprehensive perioperative assessment, individualized risk stratification tools, and proactive complication prevention strategies rather than relying solely on procedure-specific risk profiles.

## 3 The effect of immunosuppressive therapy exposure

Immunosuppressive therapy constituted a critical, disease-specific modifier of postoperative wound risk. A large National Surgical Quality Improvement Program (NSQIP) analysis of over 94,000 plastic surgery cases demonstrated that chronic steroid use independently increased both surgical and medical complications, with particularly high risk observed in flap surgery and delayed implant placement (14).

Disease-modifying antirheumatic drugs (DMARDs) exerted heterogeneous effects. In implant-based breast reconstruction, patients receiving DMARDs exhibited increased risks of necrosis, infection, deep vein thrombosis, pulmonary embolism, capsular contracture, and reoperation at both 90-day and 2-year follow-up. Notably, conventional DMARDs were associated with greater long-term implant-related complications compared with biologic agents (25). Conversely, in RA and other ADs, multivariate analyses have identified risk factors for postoperative wound complications, including advanced age, prolonged surgery, elevated preoperative white blood cell count, and specific surgical sites (e.g., foot surgery). The use of biologic or conventional DMARDs did not consistently emerge as an independent risk factor for wound complications (26). In chronic non-healing wound cohorts, controlled DMARD therapy was associated with shorter time to wound closure, suggesting that suppression of uncontrolled inflammation may, in specific contexts, facilitate healing (12). This paradox highlights the necessity of individualized risk modeling rather than uniform perioperative drug discontinuation.

A comprehensive pharmacologic review further emphasized that non-steroidal anti-inflammatory drugs (NSAIDs), corticosteroids, DMARDs, and biologics disrupted multiple stages of soft-tissue and bone healing, with no universal consensus on optimal perioperative management, reinforcing the need for structured risk stratification (27).

## 4 Disease-specific subgroup analyses: systemic lupus erythematosus vs rheumatoid arthritis and high-dose corticosteroid users

The divergent postoperative outcomes observed between SLE and RA likely reflect their distinct immunopathogenic profiles. Although patients with SLE may have a higher risk of infection and thrombosis than those with RA, this remains a hypothesis requiring further validation. In SLE, a persistent type I interferon (IFN-I) signature created a hostile environment for tissue repair by suppressing keratinocyte proliferation and migration and promoting neutrophil extracellular traps (NETs) formation. Impaired NETs clearance leaded to complement activation, immune-complex deposition, endothelial injury, and microvascular thrombosis, sustaining a pro-inflammatory and pro-thrombotic state. This milieu may result in tissue ischemia and compromised wound healing (28). Future studies should clarify the roles of immune-complex vasculopathy, complement activation, and antiphospholipid antibodies in these outcomes. Conversely, the healing impairment in RA was driven by a “TNF-α/IL-6-dominant” inflammatory milieu that sustained synovial-like inflammation (29). This environment promoted excessive MMPs production by fibroblasts, leading to premature ECM degradation, disrupted collagen deposition, and failure to progress from inflammation to effective tissue repair (30). While SLE-related risk is potential characterized by acute vascular and thrombotic events, RA-related complications are more linked to cumulative inflammatory burden and long-term remodeling failures. This mechanistic distinction may necessitate a paradigm shift: perioperative strategies for SLE should prioritize the management of vasculitic activity and complement inhibition, whereas for RA, the focus should remain on optimizing the “window” of cytokine suppression to facilitate fibroblast-mediated tissue synthesis.

Chronic or high-dose steroid exposure has represented one of the strongest predictors of postoperative wound complications across surgical fields, including infection, delayed healing, and implant failure (31). Procedures involving foreign materials or altered biomechanics, such as breast implants, arthroplasty, and spinal instrumentation, exhibited amplified complication rates in autoimmune populations, reflecting impaired host–implant interaction and chronic inflammatory responses (20, 22, 25, 31, 32).

Overall, these observations emphasize that both underlying autoimmune pathology and therapeutic exposure shape surgical risk. Adults with systemic autoimmune rheumatic diseases undergoing elective major surgery, especially implant-based procedures (e.g., arthroplasty, spinal instrumentation, breast implants), might face heightened postoperative risk, particularly with SLE and chronic steroid exposure. This proposed score focuses on stratification by disease phenotype (SLE/vasculitis-spectrum vs RA vs Spondyloarthritis) or procedure type (implant vs non-implant) to guide immunologic optimization and steroid-sparing strategies.

## 5 Future research agenda

Future research should prioritize prospective, multicenter external validation of a multidimensional postoperative wound complication risk score specifically designed for patients with ADs. Key domains should encompass disease-related factors, immunosuppressive therapy exposure, surgical complexity, and patient-specific modifiers. **Table 2** outlines candidate predictors for the Autoimmune Postoperative Wound Complication Risk Score (APWCRS), presents as a conceptual immunologic scaffold rather than a validated clinical prediction tool.

**Table 2:** Conceptual immunology-centered domains for the Autoimmune Postoperative Wound Complication Risk Score (APWCRS).

Hierarchy	Domain	Candidate variables	Conceptual rationale
Primary immune determinants	Immune activity	DAS28/CDAI (RA); SLEDAI (SLE); BASDAI/ASDAS (SpA); BVAS (vasculitis); CRP/ESR; optional future biomarkers (IL-6, TNF-α, IFN signature)	Reflects the biological intensity of immune dysregulation driving impaired healing.
Primary immune determinants	Immune-mediated vascular injury	History of vasculitis; Raynaud phenomenon; digital ulcers; antiphospholipid syndrome; complement C3/C4; anti-dsDNA (SLE)	Captures endothelial injury, thrombosis and tissue ischemia.
Primary immune determinants	Immunomodulatory therapy	Prednisone dose; csDMARDs; bDMARDs; tsDMARDs; timing of last dose	Defines perioperative immune status and host defense.
Secondary disease phenotype	Autoimmune disease subtype	RA; SLE; systemic sclerosis; vasculitis; SpA; inflammatory myopathy	Accounts for disease-specific mechanisms and wound biology.
Secondary disease phenotype	Procedure context	Implant vs non-implant surgery; contamination risk	Provides disease-context interaction rather than primary biology.
Conventional surgical modifiers	Host modifiers	Age; BMI; diabetes/HbA1c; CKD; smoking; albumin; lymphocyte count	Modify immune recovery but are not primary drivers.
Conventional surgical modifiers	Operative modifiers	ASA class; operative duration; wound class	Complement immune-based prediction by capturing surgical burden.

DAS28, Disease Activity Score in 28 joints; CDAI, Clinical disease activity index; RA, rheumatoid arthritis; SLEDAI, Systemic Lupus Erythematosus Disease Activity Index; SLE, Systemic Lupus Erythematosus; BASDAI, Bath Ankylosing Spondylitis Disease Activity Index; ASDAS, Ankylosing Spondylitis Disease Activity Score; SpA, Spondyloarthritis; BVAS, Birmingham Vasculitis Activity Score; CRP, C-Reactive Protein; ESR, Erythrocyte sedimentation rate; IL-6, Interleukin-6; TNF, Tumor Necrosis Factor; IFN, Interferon; csDMARDs, Conventional Synthetic Disease-Modifying Antirheumatic Drugs; bDMARDs, Biological Disease-Modifying Antirheumatic Drugs; tsDMARDs, Targeted Synthetic Disease-Modifying Anti-Rheumatic Drugs; vs, versus; BMI, Body Mass Index; HbA1c, Glycated Hemoglobin; CKD, Chronic Kidney Disease; ASA, American Society of Anesthesiologists.

## 6 Conclusion

Systemic autoimmune rheumatic diseases create a biologically high-risk environment for postoperative wound complications through persistent immune dysregulation, immune-mediated vascular injury, and exposure to immunosuppressive therapies. Across surgical disciplines, these conditions are associated with increased risks of infection, delayed wound healing, thromboembolic events, and reoperation, particularly in patients with SLE, vasculitis-spectrum diseases, and chronic corticosteroid exposure. The heterogeneous effects of immunomodulatory therapies further highlight the limitations of uniform perioperative management strategies. Collectively, these observations underscore the need for immunology-informed, disease-specific risk stratification frameworks to guide perioperative optimization, multidisciplinary decision-making, and future prospective investigation.
